# Observations on the Extraction of the Teeth, with Plates

**Published:** 1843-12

**Authors:** 


### Bibliographical Notices.

*Observations on the extraction of the Teeth, with plates.* By J. Chitty Clendon, Surgeon Dentist. London; S. Highley, 12mo, pp. 80, 1843.

We announced, in the June number of the third volume of the Journal, the publication of the above named work, but as we had not then seen it, we were unable to speak of its merits. We have since received and read it, and should have presented our readers with a brief expose of the author's views and the manner in which he has treated his subject, in the first number of the present volume, had we not been prevented by want of room. But although delayed until now, in performing this task, an outline of the plan of the treatise, may not, nevertheless, even at this time, be uninteresting to the profession in this country. We shall, therefore, proceed to notice such parts of it as we may deem most worthy of consideration.

The work is very well written and neatly gotten up, and the object which the author had in view in writing it, was to supply what he conceived to be a want in the literature of dental surgery. To what extent he has done this, we shall endeavor to show. That many parts of his design have been well executed, all who have read his book, will acknowledge, but he has not carried out all as fully and satisfactorily as could have been wished.

The author, in the introduction, after asserting the necessity for the extraction of such decayed and aching teeth as cannot be relieved and restored to health, very properly denounces that class of empirical practitioners who condemn the operation, and endeavor to persuade their patients that it is unnecessary, merely for the purpose of inducing them to submit to some other treatment which they may prescribe, that shall realize to themselves greater pecuniary gain. As a proof that teeth, which have become obnoxious to the living parts that surround them, should be extracted, he adduces the well known fact that the efforts of nature are, in various ways, exerted to expel them. After describing the means employed by the economy for the removal of such teeth, which consist in the filling up and destruction of their sockets, he enumerates the "pain and irritation," to which the patient, in the mean time, is subjected, and the injury that is liable to result to the contiguous parts, as reasons why the operation should at once be performed.

But while he urges the necessity of the extraction of such teeth as cannot be permitted to remain in the mouth with impunity, he is not in favor of removing any that can be made healthy and sound by filing or plugging. He at the same time contends, and in the majority of cases, he is unquestionably correct, "that when a tooth has become the seat of severe pain, the favorable period for filling the cavity has passed, and the chances of saving it very remote." There is so much truth and sound sense in this remark, we wish its practical bearing and importance, were more generally felt and acknowledged than they seem generally to be by the members of the dental profession. Teeth are filled, every day, that cannot by any surgical operation be so restored as not to be productive of irritation to the parts with which they are in contact, and which, by being thus tampered with, are rendered more offensive to them than they would otherwise have been.

The necessity for the extraction of teeth being established, the author thinks the "assumed cruelty" of the operation "disposed of, although" it may "attach to the manner in which it is too frequently performed," and in this opinion we fully coincide. Few persons, even among those who are in the daily habit of extracting teeth, perform the operation with as much dexterity and skill as they might do, were they provided with suitable instruments and properly instructed in their use.

The views of the author as expressed in the introduction, are based upon sound physiological principles, and discover him to be a man of judgment and experience.

The work is divided into three parts—the first treats on the "key instrument" and its employment for the extraction of teeth—the second on the use of forceps—and the third on the advantages of these last named instruments, to which the author gives a decided preference. After noticing the principle on which the former acts, he states that, to the application of this, "and to the use of the instrument altogether, from certain circumstances," which he does not here relate, he "early entertained a strong repugnance, a feeling which time and experience" had "increased rather than diminished." Even when placed in experienced and careful hands, he says, "the instrument is often *difficult* of *application, unscientific* and *uncertain* in its operation, and always productive of *additional pain* and *subsequent* inconvenience." He next proceeds to notice the difficulty of applying the instrument. The size of the claw, he says, must be determined by that of the tooth to which it is to be applied, which should "act in a line as nearly parallel" to the fulcrum "as the principle of a lever will admit." But we do not deem it necessary to notice the various things which he enumerates and describes as being necessary to be considered in the employment of this instrument. We will, however, quote what he says concerning the error under which very many practitioners labor with regard to the agency the claw exerts in raising a tooth from its socket.

He remarks, "to lift a tooth without doing violence to the socket in which it is placed, the necessary extractive force must be applied either in a *perpendicular* or in an *oblique* direction; the want of sufficient space and risk of injury to the antagonist teeth will generally prevent the former, and to accomplish the latter, the sweep taken must be in proportion to the length of the roots and their position in the socket. Now the edge of the claw in turning, describes the segment of a circle whose diameter is far too small to admit of the tooth being raised obliquely; the moment pressure is applied to the handle, the fulcrum turns abruptly on its bearing, and drags the claw in a parallel direction, the effect of which is to cant or upset the tooth—the claw itself preventing the former from rising over the edge of the socket."

Here the author, in a note, alludes to the directions given by Mr. Bell for the use of the key, "who," he says, "evidently feels the force of this conclusion, as he recommends a *perpendicular movement* to be given to the instrument, to facilitate the extraction of the tooth, forgetting for the moment that the success of the operation must chiefly depend on keeping the fulcrum steadily in its place; that directly it is raised, it ceases to act as a lever, and becomes in fact, only an imperfect pair of forceps."

But proceeding with the subject, he says, "by removing the tooth in this lateral direction, the natural obstructions are very much increased, and from causes, which" he thus explains:

"If we suppose the crown strong enough to sustain a force adequate to remove the tooth, the claw in the first instance will press it against that part of the alveolus on which the fulcrum rests, while the latter, by its own pressure, in a great measure counteracts the effect produced by the claw, as it not only forces the alveolus against the roots, but prevents the former from yielding, so as to afford a passage for the escape of the tooth; at the same time the extremities of the root press on the opposite side of the alveolus, which offers an effectual resistance, and it is only when the fulcrum has turned sufficiently to allow the socket to break away, that the tooth can possibly be removed. When the latter is very firmly fixed, and the crown proves unequal to bear this lateral pressure, it will assuredly break off at the neck—a consequence the more likely to ensue, as the claw, however low it may be thrust, has a tendency, from the motion of the instrument to rise until it catches the ridge formed by the terminal line of the enamel."

Mr. Clendon proves the correctness of the foregoing explanation, by showing the "effect of the claw on a stout nail driven into a piece of close grained wood—bearing some resemblance to the form of the jaw—to an extent equal to the depth of the roots in their sockets, and requiring the same degree of force to withdraw it as to extract a tooth. The fulcrum must be made to bear on the wood, and the claw to grasp the nail by a groove previously cut for that purpose. If the handle of the instrument be now turned, the claw will immediately press on the nail, and bend it to a curve corresponding with its own direction, and then, if the hold continue sufficient, it will be withdrawn. If the nail were of cast iron, or any brittle, unyielding substance, instead of bending, it would break just below the surface of the wood, immediately adequate force was used; but should it prove equal to withstand this force, the wood must then give way, or the instrument be broken."

The reasoning of the author is philosophical, and with us, had we never had any experience in the use of the key instrument, it would be conclusive. We, however, used this instrument nearly altogether, for the extraction of teeth, for many years; and although it was exceedingly seldom that we failed to perform the operation with ease and at once with it, we do not hesitate to affirm that any tooth that can be extracted with the key, can also be removed with properly constructed forceps, and that too, in the majority of instances, with greater ease to the operator, and less pain to the patient. We know that in the expression of this opinion, we differ from many of our professional brethren; and that there are many skilful and experienced practitioners who, while they prefer the forceps for the extraction of most teeth, nevertheless occasionally employ the key. We are confident that these same practitioners, if they would provide themselves with properly constructed forceps, for the extraction of those teeth which they prefer to remove with the key, and would use them for six months, to the exclusion of that instrument, they would never go back to its use again. We could mention the names of more than thirty, who, at our instance, have done this, and the result has been that all have entirely abandoned its use; and we believe the day is not distant when it will not be found among the instruments of any dentist in the land. We have not had one in our possession for nine years, and in a conversation which we, a few days since, had with an extensive manufacturer of dental and surgical instruments, he informed us that he sold fifty sets of teeth forceps to where he disposed of one key. That instrument, it is certain, is rapidly going into disuse among American dentists.

In discussing the subject of the use of this instrument, Mr. C. notices, in succession, and at some length, the "uncertain effects of the claw," the "pain produced by the pressure of the fulcrum," the "obstructions caused by the irregular form of the roots," and then concludes this part of his treatise with some general remarks.

In the second part of the work, which treats on the form, application and extraction of teeth with forceps, the author intimates that those who condemn the use of them, and have returned back to the key, have made trial of such as were as illy calculated for the purpose as "common pliers or carpenter's pincers." The defects in the form and construction of forceps have hitherto, we are convinced, prevented them from coming into general use; not one pair in five hundred are of the proper form and construction, and in view of this fact, it is a matter of wonder to us, that as many use them as do. They should not only be so constructed as to fit the necks of the several classes of teeth, but should be so bent as not to interfere with the other teeth, and to enable the operator while occupying such a position by the side of his patient, as to enable him to control his head and support the jaw, and at the same time to have such a grasp upon the instrument as to be able to exercise with convenience a perfect control over the power which it may be necessary to apply to it for the removal of the tooth. The author says, and very correctly too, that "the entire length of the instrument should not exceed six inches, with the exception of those used for the wisdom teeth," which require to be a little longer. He also recommends them to be substantially made, so as to prevent them from springing in the hand, as otherwise the moving of the instrument might be mistaken for "the yielding of the tooth." These are points which should, on no account, be overlooked in the procurement of forceps, and we have urged their importance on several occasions.

Mr. Clendon divides the teeth, with reference to their extraction, into eight classes, each of which requiring a pair of forceps specially adapted to the teeth that belong to it. They are, in the upper jaw, 1. The lateral incisors and bicuspides. 2. The central incisors and cuspidati. 3. The first and second molares of the right side. 4. The same of the left side. 5. In the lower jaw, the incisors and canini. 6. The bicuspides. 7. First and second molares, right and left sides. 8. The dentes sapientiæ of the upper and lower jaws.

The beaks or chops of the forceps employed by the author for the removal of the teeth of each of the foregoing classes, differ so little from those recommended and described in our treatise on dental surgery, that we do not deem a description of them necessary. They are such, except that neither of their handles are curved, as have been manufactured by Mr. Samuel Jackson and Messrs. Daily & Arnold of Baltimore, for the last nine or ten years.

Besides these eight pair of forceps, he says, two more are necessary for the extraction of "roots of teeth,"—one for those of the upper, and one for those of the lower jaw. He recommends that the first have "both blades [beaks] and handles slightly curved, that roots of teeth situated in the back part of the mouth may be easily reached, and that the second have the blades [beaks] bent at right angles with the handles." The beaks or blades of forceps employed for the extraction of roots of teeth, should be much longer than those used for the removal of teeth that are grasped at their neck, and they should also, as is remarked by our author, be very thin; but we cannot agree with him that the pair for the extraction of roots in the lower jaw should have their beaks bent at right angles. They can be by far more eaisily applied when bent only at an angle of about forty-five degrees with the handles. This is a point to which we have paid particular attention, having made thorough trial of both descriptions of instruments, we are enabled to speak advisedly upon the subject.

The hawk's bill forceps the author regards as decidedly objectionable, acting as the instrument does, very much upon the principle of the key, the lower blade or beak performing the office of the fulcrum, whilst the upper serves as a claw. It is also objectionable for another reason, and that is, the operator, while extracting a tooth on the left side, requires the assistance of a third person to hold the head of his patient. And while upon this subject, we would remark that the operator would have much greater control over the lower molar forceps recommended by Mr. C. if their handles were curved laterally towards him, so as to form an angle of about forty-five degrees.

With regard to the manner of extracting teeth with forceps, ample directions are given, but these are points upon which we do not deem it essential, in a notice of this sort, to dwell.

The author sets forth, in the third and last part of the work, the "advantages of forceps," but as we think their superiority will be inferred and admitted from what has already been said, we do not think it necessary to notice the arguments which he advances to establish that fact, suffice it to say, they are clear and conclusive.—*Balt. Ed.*

